# Pilot study of an online intervention for young people with a first psychotic episode: Thinkapp

**DOI:** 10.1192/j.eurpsy.2022.817

**Published:** 2022-09-01

**Authors:** C. Morales-Pillado, T. Sanchez-Gutierrez, S. Barbeito, M. Mayoral, C. Arango, L. Leon, A. Ibañez, J. Rico, A. Calvo

**Affiliations:** 1 Universidad Internacional de La Rioja, Faculty Of Health Sciences, Madrid, Spain; 2 Hospital Gregorio Marañon, Department Of Child And Adolescent Psychiatry,, Madrid, Spain; 3 Hospital Ramon y Caja, Department Of Psychiatry,, Madrid, Spain; 4 AMAFE Foundation, Amafe, Madrid, Spain

**Keywords:** First Episode Psychosis, Psychosis, Mobile intervention, schizophrénia

## Abstract

**Introduction:**

Online interventions can be a complement to maintain the long-term effectiveness of psychosocial interventions in First Episode Psychosis (FEP) that have already demonstrated their efficacy in the short and medium term (Calvo et al., 2015).

**Objectives:**

To test the effectiveness of a mobile app–based intervention (Thinkapp) to improve quality of life, functioning and symptomatology, and reduce days of admission and hospitalizations, in young people with FEP.

**Methods:**

Fourteen patients with FEP, aged 14–30, recruited from Gregorio Marañón Hospital, Ramón y Cajal Hospital and AMAFE Foundation in Madrid (Spain) received treatment as usual plus a psychoeducational intervention through a mobile app. Changes in dependent variables over the course of the intervention were assessed by means of a battery of clinical tests at baseline, 3-month and 6-month follow-up using a Wilcoxon test.

**Results:**

Of the fourteen patients included, 7 patients completed the 6-month follow-up and 8 completed the 3-month follow-up. There were significant differences in days of admission (p = 0.042) between baseline and 6-month follow-up. No significant results were observed in other clinical variables.

**Conclusions:**

The study provides preliminary data potentially related to the reduction of days of admissions.

**Disclosure:**

No significant relationships.

